# Acute effects of feeding fructose, glucose and sucrose on blood lipid levels and systemic inflammation

**DOI:** 10.1186/1476-511X-13-195

**Published:** 2014-12-16

**Authors:** Faizan Jameel, Melinda Phang, Lisa G Wood, Manohar L Garg

**Affiliations:** Nutraceuticals Research Group, School of Biomedical Sciences & Pharmacy, University of Newcastle, 305C Medical Science Building, Callaghan, NSW 2308 Australia; Centre for Asthma and Respiratory Disease, School of Biomedical Sciences & Pharmacy, University of Newcastle, Callaghan, NSW Australia; Women’s and Children’s Health Research Institute, Women’s and Children Hospital, North Adelaide, South Australia Australia

## Abstract

**Background:**

Recent studies have demonstrated a relationship between fructose consumption and risk of developing metabolic syndrome. Mechanisms by which dietary fructose mediates metabolic changes are poorly understood. This study compared the effects of fructose, glucose and sucrose consumption on post-postprandial lipemia and low grade inflammation measured as hs-CRP.

**Methods:**

This was a randomized, single blinded, cross-over trial involving healthy subjects (n = 14). After an overnight fast, participants were given one of 3 different isocaloric drinks, containing 50 g of either fructose or glucose or sucrose dissolved in water. Blood samples were collected at baseline, 30, 60 and 120 minutes post intervention for the analysis of blood lipids, glucose, insulin and high sensitivity C-reactive protein (hs-CRP).

**Results:**

Glucose and sucrose supplementation initially resulted in a significant increase in glucose and insulin levels compared to fructose supplementation and returned to near baseline values within 2 hours. Change in plasma cholesterol, LDL and HDL-cholesterol (measured as area under curve, AUC) was significantly higher when participants consumed fructose compared with glucose or sucrose (P < 0.05). AUC for plasma triglyceride levels however remained unchanged regardless of the dietary intervention. Change in AUC for hs-CRP was also significantly higher in subjects consuming fructose compared with those consuming glucose (P < 0.05), but not sucrose (P = 0.07).

**Conclusion:**

This study demonstrates that fructose as a sole source of energy modulates plasma lipids and hsCRP levels in healthy individuals. The significance of increase in HDL-cholesterol with a concurrent increase in LDL-cholesterol and elevated hs-CRP levels remains to be delineated when considering health effects of feeding fructose-rich diets.

**Registration number for clinical trials:**

ACTRN12614000431628

## Introduction

Fructose, commonly known as fruit sugar, is also a major component of sweeteners such as table sugar, honey and high fructose corn syrup (HFCS). Fructose intake has quadrupled since the beginning of 20th century, partly because of the introduction of HFCS [1]. Increased fructose consumption can lead to a parallel rise in cardiovascular disease risk factors, i.e. increase in blood lipids [2, 3], development of insulin resistance [4, 5], alteration in the production of satiety hormones (insulin, leptin and ghrelin) [3], increase in inflammatory biomarkers [6, 7] and increase in obesity [5, 8]. Since similar effects do not occur following the intake of starch or glucose, it has been proposed that fructose-induced metabolic changes are not mediated by excessive sugar intake in general, but are specific to fructose. Precise underlying mechanisms by which fructose consumption may induce negative metabolic effects are not clear. One recent study demonstrated that in young healthy individuals, consumption of glucose and fructose drinks resulted in markedly different hemodynamic responses, with fructose stimulating a sustained increase in blood pressure [9]. These observations support the concept that diets with repeated fructose loads may, over time, contribute to increased cardiovascular disease risk.

The aim of this study was to investigate the effects of fructose compared to glucose and sucrose consumption, on postprandial lipemia and low grade inflammation in healthy subjects. Previous studies have examined the effects of feeding sugars as part of a meal on cardiovascular disease indices. In the current study, we looked at postprandial lipid and low grade inflammation following a single dose of sugary drink given as a sole source of energy after an overnight fast.

## Methods

### Study population

Healthy male and female adults (n = 14) between the ages of 18-60 years were recruited by advertisement and underwent study procedures at the Nutraceuticals Research Group Clinic rooms, University of Newcastle, NSW, Australia. Exclusion criteria were: diagnosed hyperlipidaemia, diabetes, gastrointestinal disorders, currently on fructose/sugar restricted diet, vegan diet or weight loss program, undergone any surgical procedure for obesity, pregnant or lactating mother, taking lipid-lowering or anti-inflammatory drugs and BMI >30 kg/m^2^. Participants were asked to complete a medical questionnaire, International Physical Activity Questionnaire (IPAQ) [10] and a 24 hr food record. Approval for the study was granted by the Human Research Ethics Committee of the University of Newcastle, Australia. All participants provided written informed consent and the study was conducted in accordance with The Declaration of Helsinki. The trial was registered with the Australian & New Zealand Clinical Trials Registry (ACTRN12614000431628). Body composition was assessed by bioimpedance analysis (BIA) using single frequency bioelectrical impedance apparatus (Maltron International, Essex, UK). Measurements were conducted in the supine position, with participants wearing light clothing and without shoes, in the morning after a minimum 10 hour fast. Participants were asked to refrain from physical exertion and alcohol consumption for 24 hours prior to testing.

### Study design

The trial was a randomised, single blinded, controlled cross-over intervention trial. Participants visited the research facility on three separate occasions, where they consumed one of 3 different isocaloric sugary drinks on each occasion, with a minimum of one week wash out period in between. The participants were randomised to consume: 1) 50 g fructose dissolved in 300 ml of water 2) 50 g glucose dissolved in 300 ml of water and 3) 50 g sucrose dissolved in 300 ml of water. Each sugar drink contained 10 ml of lemon juice to provide a more uniform and palatable taste. Participants were asked to consume the sweetened drinks within 2-3 minutes and compliance was observed. Block randomization technique was used for allocation of participants to treatment arms. During each visit, a fasting blood sample was collected prior to supplementation, then 30, 60 and 120 minutes following intake of the sugary drink. The participants remained in the research facility until the final sample was collected and were asked to limit physical activity during their time in the research facility.

### Laboratory methods

Blood samples at base line, 30, 60 and 120 minutes were collected into tubes pre-coated with EDTA, lithium heparin and sodium fluoride by venepuncture. EDTA blood tubes were centrifuged for 10 minutes at 3000 g at 4°C for separation of plasma and stored at -80°C for further use. The lithium heparin tubes for blood lipids and sodium fluoride tubes for blood glucose and insulin measurement were analysed by Hunter New England Area Pathology Services (Newcastle). Twenty four hour food recalls were collected by face to face interview by an in-house dietician. Food records collected from participants were entered into FoodWorks Version 7.0.291 database (Xyris Software Pty Ltd, Queensland, Australia) to analyse daily energy and nutrient intake of participants.

### Statistical analysis

All data are presented as mean ± SEM. Area under the curve (AUC) was calculated by the trapezoid method. Preliminary assumption testing was done to check for normality, linearity, outliers and homogeneity of variance with no serious violations noted for all test variables within the three different groups of fructose, glucose and sucrose. Comparisons between different groups were made with one-way repeated measures ANOVA and post hoc Tukey testing. A probability level of p < 0.05 was adopted throughout to determine statistical significance unless otherwise mentioned. All statistical analyses were carried out with SPSS software (version 21.0; SPSS Inc., Chicago, IL, USA).

## Results

All participants were healthy and their baseline values of anthropometric measurements and blood biomarkers were within normal range (Table 1).

**Figure 1:**
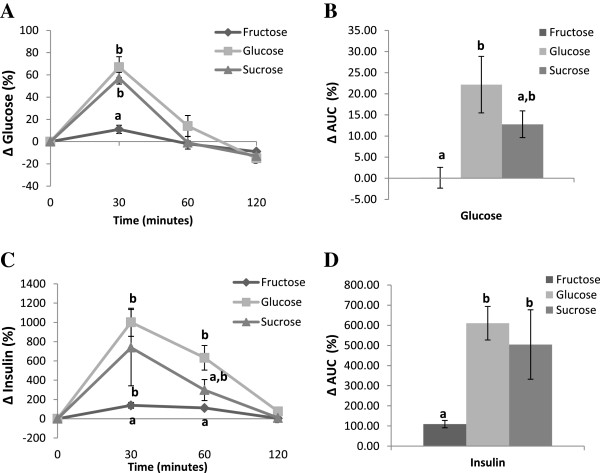
**Kinetics of change and area under the curve (AUC) for blood glucose (mmol/L) (A and B respectively) and insulin (mIU/L) (C and D respectively) after the fructose, glucose or sucrose supplementations.** Values without a common superscript are significantly different; P < 0.05.

As shown in Figure 1A, at 30 minutes, fructose consumption was followed by an increase in blood glucose levels that was significantly lower than following glucose and sucrose consumption. At 60 and 120 minutes there were no differences between the 3 groups. The overall change in blood glucose levels followed by fructose consumption measured as area under the curve (AUC), was significantly lower than following glucose supplementation (Figure 1B).

Figure 1C demonstrates that after 30 minutes, fructose consumption led to a smaller increase in insulin levels than glucose or sucrose. At 60 minutes, insulin levels in the fructose fed group remained lower than the glucose fed group. However, at 120 minutes, there were no differences in insulin levels across intervention groups. The overall increase in the insulin levels, measured as AUC, was significantly lower when participants consumed fructose compared to glucose or sucrose (Figure 1D).

**Table 1:** **Baseline values of anthropometric measurements, blood biomarkers & energy and nutrients intakes of study participants**

A. Baseline characteristics			
	Total	Males	Females
n	14	07	07
Age (Yrs)	28.0 ± 0.79	28.7 ± 0.64	27.3 ± 1.45
Weight (kg)	69.6 ± 3.48	78.8 ± 4.26	60.4 ± 2.50
BMI (kg/m^2^)	24.2 ± 0.72	25.6 ± 0.82	22.8 ± 0.98
SMM (kg)	30.8 ± 2.44	34.1 ± 2.72	27.4 ± 3.83
FFM (kg)	51.1 ± 3.48	60.0 ± 4.44	42.1 ± 2.49
PBF (%)	27.1 ± 1.88	24.0 ± 1.86	30.1 ± 2.96
Waist: hip	0.8 ± 0.00	0.9 ± 0.00	0.8 ± 0.12
Glucose (mmol/L)	4.7 ± 0.12	4.7 ± 0.19	4.6 ± 0.16
Cholesterol (mmol/L)	4.2 ± 0.22	4.3 ± 0.37	4.1 ± 0.25
Triglyceride (mmol/L)	1.01 ± 0.20	1.35 ± 0.35	0.67 ± 0.11
LDL-C (mmol/L)	2.43 ± 0.22	2.72 ± 0.39	2.14 ± 0.19
HDL-C (mmol/L)	1.32 ± 0.13	1.00 ± 0.09	1.65 ± 0.19
Total/HDL ratio	3.6 ± 0.38	4.5 ± 0.52	2.6 ± 0.25
Insulin (mIU/L)	9.2 ± 1.09	9.5 ± 1.56	8.8 ± 1.65
CRP (mg/L)	1.5 ± 0.31	1.7 ± 0.47	1.5 ± 0.45
**B: Daily energy and nutrient intake**			
Daily intake	Total	Males	Females
Energy No DF (kj)	8619 ± 1001	10077 ± 1797	7161 ± 641
Energy DF (kj)	8820 ± 1016	10292 ± 1828	7348 ± 645
Protein (g)	89.37 ± 11.78	107.82 ± 21.00	70.92 ± 6.86
Fat (g)	73.29 ± 10.20	90.56 ± 16.78	56.03 ± 8.99
Cholesterol (mg)	204.46 ± 43.01	227.38 ± 81.43	181.53 ± 34.81
Carbohydrate (g)	481.54 ± 224.56	293.89 ± 58.01	669.18 ± 451.01
Sugar (g)	88.21 ± 12.24	110.05 ± 21.72	76.20 ± 11.41
Glucose (g)	10.40 ± 2.32	9.54 ± 4.27	11.27 ± 2.19
Sucrose (g)	32.70 ± 9.06	35.50 ± 17.15	29.90 ± 7.67
Bound fructose (g)	16.35 ± 4.53	17.75 ± 8.57	14.95 ± 3.83
Free fructose (g)	12.56 ± 2.27	11.60 ± 3.93	13.51 ± 2.57
Total fructose (g)	28.91 ± 6.51	29.55 ± 12.36	28.46 ± 5.56
Lactose (g)	12.36 ± 2.09	16.13 ± 2.92	8.60 ± 2.40
Maltose (g)	18.01 ± 5.84	25.74 ± 10.06	10.29 ± 5.15

Mean values ± standard error of mean.

BMI, Body mass index; SMM, Skeletal muscle mass, FM, Fat Free Mass; PBF, Percentage Body Fat; LDL-C, Low Density Lipoprotein cholesterol; HDL-C, High Density Lipoprotein cholesterol; CRP, c-Reactive Protein, DF: dietary fibre.

**Figure 2:**
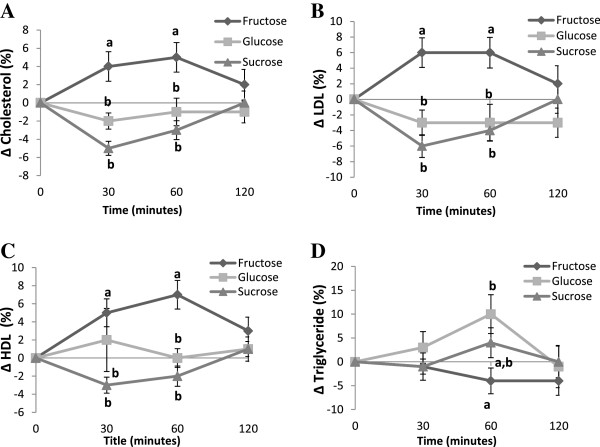
**Kinetics of change for (A) total cholesterol (mmol/L); (B) LDL-C (mmol/L); (C)HDL-C (mmol/L) and (D) triglyceride (mmol/L) after the fructose, glucose or sucrose supplementations.** Values without a common superscript are significantly different; P < 0.05.

Fructose consumption led to an increase in total cholesterol compared to glucose and sucrose consumption (Figure 2A). The effects were still apparent at 60 minutes, however at 120 minutes there were no differences between groups. The overall increase in plasma total cholesterol measured as AUC was significantly higher when participants consumed fructose compared to glucose or sucrose (Table 2). At 30 minutes after fructose consumption we observed an increase in LDL-cholesterol compared to glucose and sucrose (Figure 2B). The effects were still apparent at 60 minutes. However, at 120 minutes there were no differences between groups. Overall, the increase in LDL cholesterol measured as AUC was significantly higher when participants consumed fructose compared to glucose or sucrose (Table 2). Similarly, fructose consumption was followed by an increase in HDL-cholesterol at 30 minutes in comparison to glucose and sucrose (Figure 2C). The effects were still apparent at 60 minutes; however, at 120 minutes there were no differences between groups. The overall increase in HDL cholesterol measured as AUC was significantly higher when participants consumed fructose compared to glucose or sucrose (Table 2).

**Table 2:** **Area under curve (AUC) for blood lipids derived from kinetics of change**

Area under curve (AUC)			
	Fructose (F)	Glucose (G)	Sucrose (S)
Cholesterol (mmol/L)	4.09 ± 1.67^a^	-0.90 ± 1.25^b^	-2.41 ± 0.85^b^
LDL-C (mmol/L)	4.50 ± 2.03^a^	-3.22 ± 1.69^b^	-3.87 ± 1.25^b^
HDL-C (mmol/L)	6.69 ± 1.56^a^	0.63 ± 1.70^b^	-0.84 ± 0.60^b^
Triglyceride (mmol/L)	-2.45 ± 2.37	5.65 ± 3.52	1.79 ± 2.68
Total/HDL ratio	-1.97 ± 0.92	-0.62 ± 0.73	-1.57 ± 0.95

Mean values± standard error of mean; LDL-C, Low Density Lipoprotein cholesterol; HDL-C, High Density Lipoprotein cholesterol; Values without a common superscript are significantly different, P<0.05.

Comparison of all treatment groups revealed no significant difference in TG levels at 30 minutes. At 60 minutes, subjects fed fructose had lower TG levels compared to glucose. At 120 minutes there were no differences between groups (Figure 2D). There were overall no significant differences in plasma triglyceride levels (measured as AUC) regardless of the dietary intervention (Table 2). Comparison between all treatment groups revealed no significant change in the ratio of total/HDL-cholesterol at all the time points (p > 0.005). Furthermore, the overall change in the ratio of total/HDL-cholesterol measured as AUC was not different between groups (Table 2).

**Figure 3:**
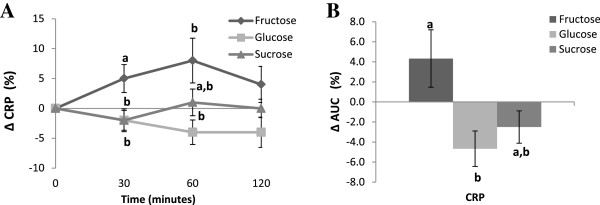
**Kinetics of change (A) and area under the curve (AUC) (B) for CRP (mg/L) after the fructose, glucose or sucrose supplementations.** Values without a common superscript are significantly different; P < 0.05.

Figure 3A demonstrates that fructose consumption was followed by an increase in hs-CRP level at 30 minutes when compared to glucose and sucrose. At 60 minutes, hs-CRP was not different compared to glucose or sucrose and at 120 minutes there were no differences between groups. There was, however, an overall increase in hs-CRP levels measured as AUC in subjects who consumed fructose compared with those who consumed glucose (p < 0.05), but not sucrose (Figure 3B).

## Discussion

This study was designed to examine the metabolic consequences of sugar consumption when it is used as a sole source of energy. Participants consumed fructose in the fasting state in the morning; therefore, majority of the sugar consumed would be used to produce energy and/or partly used to replenish glycogen stores at this time of day [11]. Fructose was a sole source of energy without accompanying meal and there was no other nutrient to augment postprandial lipemia. Consumption of sugar sweetened beverages in the morning in the fasting state separated the effect of fructose from excess energy intake which may be a confounder in the overfeeding studies [11]. This is the first study to report the consequences of consuming a beverage containing fructose, glucose or sucrose as a sole source of energy, on postprandial lipid levels and inflammation markers. Acute fructose consumption in a single dose of 50 g/day which provided approximately 8% of daily energy in the form of a beverage, resulted in a significant increase in the plasma levels of total, LDL and HDL cholesterol and the acute phase pro-inflammatory marker hs-CRP, compared to the same dose of glucose or sucrose. Interestingly, no significant change in TG levels was observed.

The change in fasting glucose and insulin responses was modest in fructose compared with glucose and sucrose groups. This modest increase in the glucose and insulin levels after fructose consumption is consistent with previous studies [12–14]. The blunted rise in insulin in response to fructose consumption is consistent with the blunted rise in blood glucose level, but may also be partly attributed to less release of intestinal incretin hormone that binds to β-cells of the pancreas leading to reduce secretion of insulin [15].

Changes in total, LDL and HDL-cholesterol levels were significantly higher when participants consumed fructose compared with glucose or sucrose sweetened beverage. Previous studies examining postprandial lipemia following fructose consumption were either focused on triglyceride levels only [11, 14, 16–19] or demonstrate heterogeneous results. Two studies showed no effects of fructose on plasma total, LDL or HDL-cholesterol levels [20, 21], while another 2 studies [2, 22] showed a significant increase in fasting serum total and LDL cholesterol following 4-5 weeks of consuming fructose-rich diets compared to the starch diet. The reason for the increase in postprandial levels of total, LDL and HDL cholesterol in subjects who consumed fructose in our study is not known. Since no nutrients, other than sugars, were included in the test beverages, the lipoproteins measured were almost exclusively of hepatic origin. Whether fructose can influence total and LDL-cholesterol levels by blocking LDL-receptors or affect HDL cholesterol via CETP or reverse cholesterol transport is not known, therefore, merits further investigation.

Dietary fructose has been previously shown to cause either no change [2, 21, 23, 24] or an increase in fasting plasma triglycerides in healthy subjects [20, 25]. Our results showed no significant change in postprandial triglyceride levels irrespective of the type of sugar. These findings are in agreement with a meta-analysis reporting no significant change in post-prandial TG level unless the amount of fructose exceeds 50 g/day [26]. On the other hand, the present results are in contrast with literature reporting an increase in TG after acute fructose consumption [3, 11, 14, 16–18, 25, 27]. This disagreement may be due to the accompanying meal with consumption of the fructose beverage in previously published studies. In our study, due to the absence of other energy yielding nutrients, the clearance rate of triglycerides can be expected to be higher, resulting in overall no change. Moreover the shorter duration (2 hours) of our study compared to the study by Bohannan et al. [19] (5 hours) may account for the discrepancy in the two studies. Therefore, the lipemic effects of fructose may depend on the dose and duration of fructose feeding and whether fructose is consumed in the presence or absence of other energy nutrients.

The pro-inflammatory biomarker that we examined in this study was hs-CRP. The area under the curve for hs-CRP level was significantly increased in the fructose group compared with glucose, but not with sucrose. This is the first time, to our knowledge, in healthy and normal weight adult subjects, that acute fructose consumption has been shown to increase hs-CRP levels. Previous studies reporting an increase in hs-CRP level were either conducted in a mixed population of lean and overweight individuals [12] or a long term intervention study [6]. The proposed mechanism of fructose-induced oxidative stress and inflammation markers (TNFα, IL-6, IL-1β) [28] potentially resulting in an increase synthesis of hs-CRP merits further investigation. Fructose has been shown to induce oxidative stress in cellular [29] and animal models [30], thereby, may result in elevated levels of pro-inflammatory mediators. Whether fructose can directly (without conversion to fat) influence inflammation pathways (leukotriene synthesis, expression of adhesion molecules etc) remains to be delineated. In conclusion, this study demonstrates that fructose as a sole source of energy modulates plasma lipids and hsCRP levels in healthy individuals. However, the significance of increase in HDL-cholesterol with a concurrent increase in LDL-cholesterol and elevated hsCRP levels remains to be delineated when considering health effects of feeding fructose-rich diets.
